# Influenza‐associated respiratory illness among five cohorts of pregnant women and their young infants (0–6 months), Bangladesh, 2013–2017

**DOI:** 10.1111/irv.13175

**Published:** 2023-08-13

**Authors:** Zubair Akhtar, Probir Ghosh, Mejbah Bhuiyan, Katharine Sturm‐Ramirez, Mohammed Rahman, Md. Howlader, Fatimah Dawood, Fahmida Chowdhury, Danielle Iuliano

**Affiliations:** ^1^ International Centre for Diarrhoeal Disease and Research, Bangladesh (icddr,b) Dhaka Bangladesh; ^2^ GSK vaccines Siena Italy; ^3^ Centers for Disease Control and Prevention (CDC) Atlanta Georgia USA; ^4^ US Public Health Service (USPHS) Washington District of Columbia USA

**Keywords:** 0–6 months infant influenza, LMP, maternal influenza, portable ultrasound, pregnancy

## Abstract

**Background:**

Pregnant women with their infants are considered at higher risk for influenza‐associated complications, and the World Health Organization (WHO) recommends influenza vaccination during pregnancy to protect them, including their infants (0–6 months). There are limited data on the influenza burden among pregnant women and their infants (0–6 months), and there are no routine influenza vaccinations in Bangladesh.

**Methods:**

Five annual cohorts (2013–2017) of pregnant women were enrolled from the eight sub‐districts of Bangladesh before the influenza season (May–September); they were contacted weekly to identify new onset of influenza‐like illness (ILI) (subjective or measured fever and cough) and acute respiratory illness (ARI) (at least two of these symptoms: cough, rhinorrhea, or difficulty in breathing) among their infants from birth to 6 months of age. We collected nasopharyngeal swabs from ILI and ARI cases, tested by real‐time reverse transcription polymerase chain reaction (rRT‐PCR) for influenza virus (including types and subtypes) and estimated influenza incidence (95% CI)/10000 pregnant women‐months or infant‐months, respectively.

**Results:**

We enrolled 9020 pregnant women, followed for 26,709 pregnancy‐months, and detected 1241 ILI episodes. We also followed 8963 infants for 51,518 infant‐months and identified 5116 ARI episodes. Influenza positivity was 23% for ILI and 3% for ARI cases. The overall incidence (2013–2017) of influenza among pregnant women was 158.5/10000 pregnant women‐months (95% CI: 141.4–177.6) and that among infants was 21.9/10000 infant‐months (95% CI: 18.2–26.5).

**Conclusions:**

Although the data was collected more than 5 years ago, as the only baseline data, our findings illustrate evidence of influenza burden among pregnant women and infants (0–6 months), which may support preventive policy decisions in Bangladesh.

## 1 INTRODUCTION

Epidemiological studies have shown that pregnant women are at higher risk for influenza‐associated hospitalizations during annual seasonal epidemics^1^ and have an increased hospitalization rate for respiratory diseases during the annual influenza season than non‐pregnant women.^2^ In light of clear evidence of the risks associated with influenza virus infection among pregnant women, the World Health Organization's (WHO) strategic advisory group of experts (SAGE) working group considers pregnant women as a high priority risk group and has recommended influenza vaccination.^3^

Postpartum women and infants <6 months are also at increased risk of severe influenza virus infections.^4^, ^5^ Because infants <6 months of age cannot be vaccinated against influenza, the risk of severe illness due to influenza virus infection remains high.^6^ A study from Mongolia from 2013 to 2015 showed that 7.4% of infants <6 months of age had influenza virus infections.^7^ A previous study in Bangladesh in 2009 showed a high incidence (6/100 child years) of influenza virus infection among infants <6 months of age.^8^

Annual influenza vaccination has been an important measure of preventing and controlling influenza virus infections.^9^ Influenza vaccination during pregnancy was reported to reduce the incidence of influenza illnesses and hospitalizations among infants <6 months of age.^10^, ^11^ However, in most low‐income countries, including Bangladesh, the influenza vaccine is not part of routine immunization practices,^12^ and pregnant women are unlikely to be vaccinated.

There are limited data on the burden of laboratory‐confirmed seasonal influenza illnesses among pregnant women and their young infants in Bangladesh.^13^ These data are helpful for policymakers to consider future control and prevention strategies against influenza like surveillance, non‐pharmaceutical interventions, and vaccination. This study estimated the incidence of influenza virus infection among pregnant women and their infants <6 months of age during the five influenza seasons in four administrative districts of Bangladesh.

## 2 METHODS

### 2.1 Study population and setting

**FIGURE 1:**
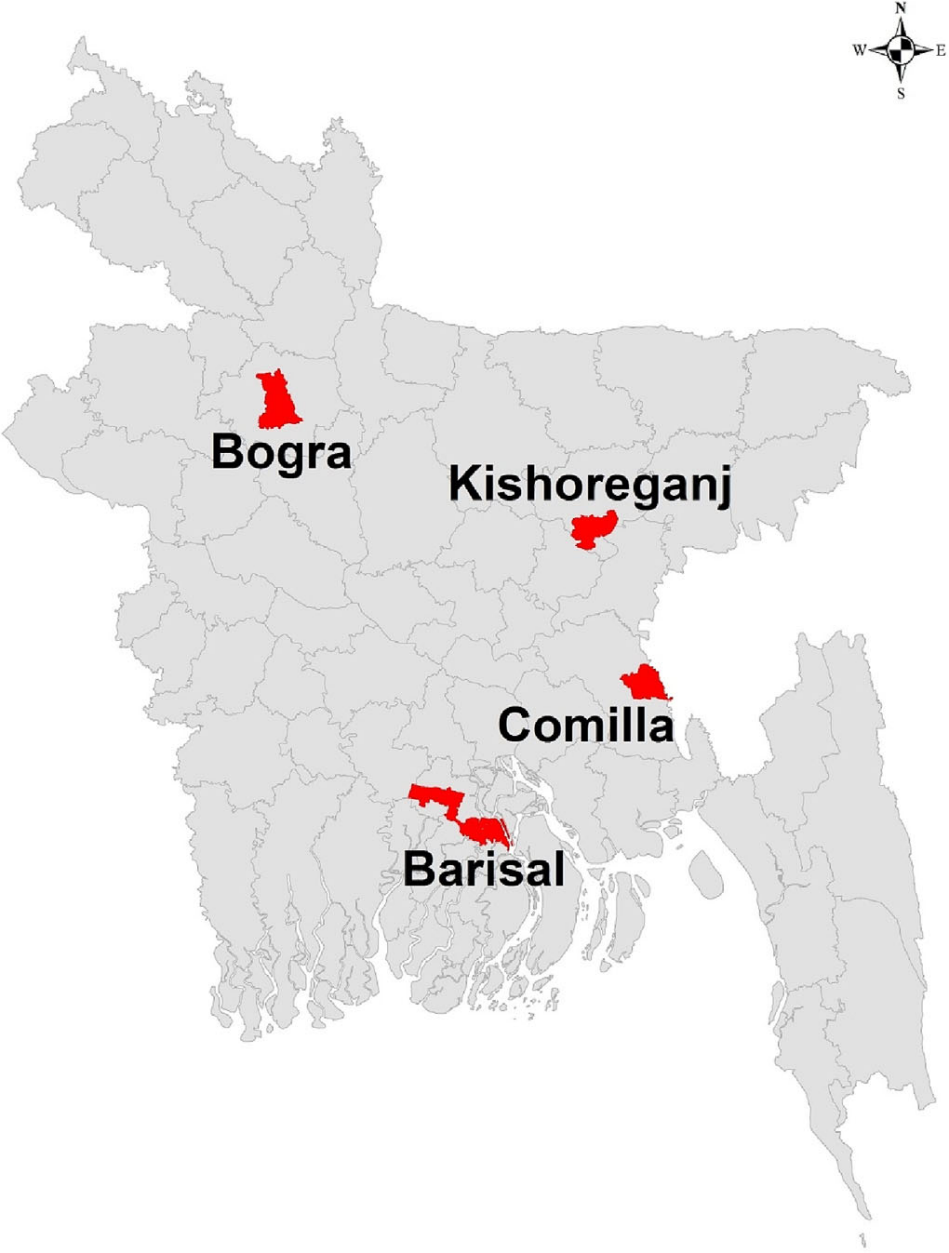
Study sites in four districts of Bangladesh, 2013–2017.

From 2013 to 2017, five cohorts of pregnant women of any gestational age in urban and peri‐urban areas of Cumilla, Bogura, Kishoreganj, and Barishal districts of Bangladesh were enrolled, followed until delivery. Then, their infants were followed from birth to 6 months of age to detect influenza virus infections (Figure 1).

### 2.2 Study design and sampling strategy

We conducted a prospective cohort study that enrolled pregnant women annually during March and April before the start of the influenza season, typically from May to September.^14^ To formulate the enrollment strategy, we reviewed information about the use of antenatal care (ANC) services in the four districts selected. Five pregnant women visited an ANC facility each day at the eight local sub‐district health facilities for ANC services. Therefore, we considered 2 months of enrolment before the start of the influenza season, targeting to enroll 2080 (equivalent to five pregnant women times eight sub‐district health facilities times 26 days per month for 2 months) pregnant women from eight sub‐district health facilities. We also sought to enroll another 500 pregnant women from the Government Family Planning Department's register list. We assumed 15% of refusal from the women visiting the sub‐district health facility and 10% loss of follow‐up of all enrolled women during the study period and accounted for those assumptions. Considering these assumptions, we formulated our sampling strategy of 2000 pregnant women per cohort per year, and it would yield an influenza incidence of laboratory‐confirmed infection among symptomatic pregnant women in Bangladesh.

For the first cohort, the enrollment of 2000 pregnant women was delayed and occurred from May to June 2013, after the IRB approval. We recruited the subsequent cohorts (2014–2017) using the Government Family Planning Department's register from the catchment area of eight local (sub‐district) health complexes. Leveraging this register, we sought to enroll 2000 pregnant women by April of each year. As there was no routine influenza vaccination in Bangladesh,^12^ all our study participants were likely to be unvaccinated. We then followed up on enrolled cohorts of pregnant women from 2013 to 2017 through the end of pregnancy, irrespective of live birth. We followed infants born to these pregnant women from birth to 6 months. Regardless of the pregnancy's gestational age, we requested all pregnant women to participate and enroll. Written informed consent to participate in the study was obtained from the participant or the infant's mother before enrolling, data collection, or specimen collection. The study was approved by the icddr,b institutional review board (IRB) before enrolling participants. Centers for Disease Control and Prevention (CDC)'s Human Research Protection Office has reviewed and approved the reliance on icddr,b's IRB.

### 2.3 Case definitions, participant follow‐up, data collection, and biological specimen testing

We adopted case definitions from the influenza‐like illness (ILI) case definition of the WHO.^15^ For pregnant women, a case definition of ILI was defined as subjective or measured fever and cough in the previous 7 days. For infants, a case definition of acute respiratory illness (ARI) was defined as the new onset of at least two signs or symptoms: cough, rhinorrhea, or difficulty breathing during the last 7 days.

After enrollment, we collected socio‐demographic and socioeconomic information using a structured questionnaire. Gestational age was measured using the date of the last menstrual period (LMP). Due to participants' potential recall bias about the LMP, we piloted the feasibility of use of a portable ultrasound machine in community settings. With limited resources, in 2016 and 2017, we sought to perform portable transabdominal ultrasounds among a subset of pregnant women at <24 weeks gestation as determined previously by LMP.^16^ Therefore, with resources available, this ultrasound could be performed in a community setting to have more accurate data.

To capture infant birth weight and enroll infants, the study staff conducted a household visit within 3 days following birth. From 2013 to 2015, the study staff measured birth weight with digital household scales with measurement precision to the nearest 100 g using a standard operating procedure. For the 2016–2017 cohorts, study staff registered the birth weight using baby scales (beurer BY 80), which measured weight to the nearest 5 g. These baby scales were available locally and had automatic and manual hold functions that provided more accurate birth weight measurements. For births in medical facilities, we obtained infant birth weight from birth certificates. Birth weight of <2500 g was considered a low birth weight (LBW).^17^ We defined stillbirth as a fetal death occurring after ≥28 weeks of gestation.^18^

Follow up began coinciding with the influenza season, and it was conducted once weekly with mobile phone calls or home visits if the participant was unreachable by phone. Study team had to ensure that they have contacted respondents either by phone or home visit every work day so that there were no loss to follow up unless the participant refused to continue participating in the study. Infants were followed once per week from birth to 6 months of age. Each week, we asked pregnant women if they experienced a new ILI and met the ILI case definition; a household visit was scheduled for the same day to collect detailed illness information and a nasopharyngeal (NP) swab. We considered a repeat ILI episode if the symptom onset dates of the episodes were more than 14 days apart. However, we did not consider any definite period of resolved symptoms.

Similarly, for infants, each week, we asked mothers if their infants had experienced any new respiratory signs or symptoms since the last follow‐up. We asked the mothers if their infant suffered an ARI and met the ARI case definition; we scheduled a household visit on the same day to collect detailed illness information and an NP swab. We considered a repeat ARI episode if symptom onset dates between the episodes were more than 14 days apart.

The NP swabs were stored in a viral transport media, kept inside nitrogen dry‐shippers, and transported to the virology laboratory at icddr,b in Dhaka (capital city) every 2 weeks for influenza virus testing (both type and subtype testing for A and lineage testing for B) by real‐time reverse transcription polymerase chain reaction (rRT‐PCR) using primers and probes from US CDC.

We considered a loss to follow up if a respondent withdrew consent or migrated out of the study catchment area, age verification of a pregnant woman of ≥18 years was not possible, or the termination of pregnancy by any means before the commencement of follow‐up.

### 2.4 Data analysis

We summarized demographic information for pregnant women and infants using descriptive statistics. We used principal component analysis to calculate the participant wealth index using data on economic statuses, durable asset ownership, access to utilities and infrastructure, and housing characteristics. We analyzed additional demographic and pregnancy characteristics such as parity and gestational age for their association with having an influenza virus infection. We compared gestational age obtained by LMP and ultrasound of the subset of the same respondents and used a paired *t*‐test to analyze differences in results obtained by the two methods.

We described circulating influenza viruses by type and subtype, reported ILI and influenza symptoms, the clinical course of illness episodes, and the number of individuals with repeat ILI/ARI episodes. We summarized the information regarding illness symptoms for pregnant women by ILI episodes and influenza virus infection. Similarly, we summarized illness information for infants by ARI episode and influenza virus illness.

**FIGURE 2:**
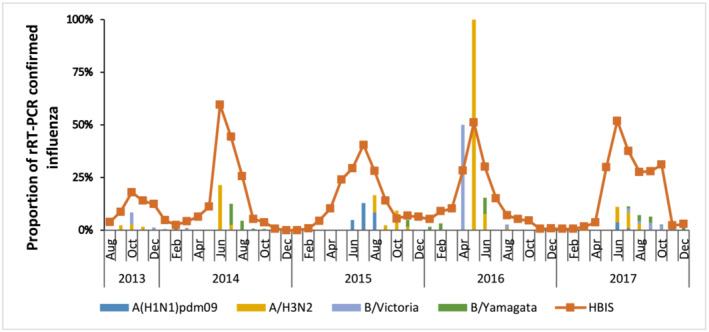
Influenza virus infection among infants in eight sub‐districts of Bangladesh, 2013–2017.

We analyzed influenza virus illness incidence among pregnant women and infants <6 months of age by influenza subtype. We calculated the incidence of influenza virus infection among pregnant women by dividing the total number of infections by the total person‐time of follow‐up in years during the peak influenza season in Bangladesh (May to September). Although infants were followed through 6 months of age regardless of when birth and influenza season occurred, only 24% of infants were born during the peak influenza season. Thus, most of the infant follow‐up time (51%) occurred outside the influenza season. Furthermore, influenza virus infection was rare outside the influenza season (Figure 2). The incidence of influenza‐virus infection for infants was therefore calculated for the person time follow‐up for May to September each year. We calculated the incidence of influenza virus infection among infants each year and divided the total number of influenza virus infections by the total infant years of follow‐up during the peak influenza season. We calculated follow‐up person‐time by summing the total follow‐up time for participants in each cohort by year and then converted it into pregnant women‐month time and infant‐month. We performed Poisson regression to estimate incidence rates (IR) with 95% confidence intervals (CI). Like previous studies,^19^ the incidence calculated excluded the 7 days following the ILI or ARI episode identification. We considered those 7 days as days of no risk of possible new influenza infection and did not include the person‐time denominator.

## 3 RESULTS

There were 10,000 pregnant women enrolled during the five‐year study period; of whom, we lost to follow‐up 980 (10%), resulting in following up of 9020 pregnant women. There were 1765/2000 (88%) in 2013, 1685/2000 (84%) in 2014, 1786/2000 (89%) in 2015, 1843/2000 (92%) in 2016, and 1941/2000 (97%) in 2017 pregnant women who completed the follow‐up (Table 1). The most common reason for the loss to follow‐up of 980 (980/10,000, 10%) pregnant women was migration out of the catchment area (*n* = 548, 56%) followed by birth before follow‐up initiation at the start of the influenza peak season (*n* = 228, 23%), respondents' whose age could not be verified or was <18 years of age (*n* = 168, 17%), and withdrawal of consent (*n* = 36, 4%) by respondents. Among the 9020 enrolled women, there were 8859 (8859/9020, 98%) live births. We enrolled 9087 infants, including 8859 infants of women who were followed under surveillance and 228 additional births that occurred before follow‐up. Of enrolled infants, 8735 (8735/9087, 96%) were followed through 6 months of age. Among the 352 infants lost to follow‐up, the most common reasons for loss to follow‐up included infant death, including neonates (*n* = 187, 53%), or migration out of the study catchment area (*n* = 165, 47%).

**TABLE 1:** Socio‐demographic characteristics and gestational age of pregnant women in eight sub‐districts of Bangladesh, 2013–2017.

Characteristics of pregnant women	Pregnant women cohort					
	2013 (*n* = 1765)	2014 (*n* = 1685)	2015 (*n* = 1786)	2016 (*n* = 1843)	2017 (*n* = 1941)	2013–17 (*n* = 9020)
Age (years)						
18–24	1113 (63.1)	1037 (61.5)	1104 (61.9)	1165 (63.2)	1154 (59.5)	5573 (61.8)
25–34	606 (34.3)	598 (35.5)	627 (35.1)	625 (33.9)	731 (37.7)	3187 (35.3)
35–44	46 (2.61)	50 (3.0)	54 (3.0)	53 (2.9)	56 (2.9)	259 (2.9)
Education						
No education	114 (6.5)	129 (7.7)	97 (5.4)	88 (4.8)	76 (3.9)	504 (5.6)
Primary education incomplete	166 (9.4)	191 (11.3)	194 (10.9)	181 (9.8)	207 (10.7)	939 (10.4)
Primary education complete	252 (14.3)	221 (13.1)	258 (14.5)	249 (13.5)	249 (12.8)	1229 (13.6)
Secondary education incomplete	886 (50.2)	803 (47.7)	883 (49.5)	905 (49.1)	943 (48.6)	4420 (49.0)
Secondary education complete or higher	347 (19.7)	341 (20.2)	353 (19.8)	420 (22.8)	466 (24.0)	1927 (21.4)
Wealth index^a^						
Poorest	354 (20.1)	334 (20.0)	352 (19.7)	405 (22.0)	410 (21.1)	1855 (20.6)
Second	352 (20.0)	314 (18.7)	363 (20.6)	350 (19.0)	368 (19.0)	1747 (19.4)
Middle	367 (20.8)	351 (20.8)	356 (20.0)	373 (20.1)	397 (20.5)	1844 (20.5)
Fourth	347 (19.7)	345 (20.5)	369 (20.7)	346 (18.8)	400 (20.6)	1807 (20.0)
Wealthiest	344 (19.5)	340 (20.2)	344 (19.3)	368 (20.0)	366 (18.9)	1762 (19.6)
Parity						
1	609 (34.5)	542 (32.2)	610 (34.2)	606 (32.9)	638 (32.9)	3005 (33.3)
2	563 (31.9)	544 (32.3)	581 (32.6)	375 (36.6)	651 (33.5)	3014 (33.4)
3	359 (20.3)	356 (21.1)	355 (19.9)	367 (20.0)	401 (20.7)	1838 (20.4)
4 or more	234 (13.3)	243 (14.4)	239 (13.4)	194 (10.5)	251 (12.9)	1161 (12.9)
Pregnancy trimester at enrollment						
1st	59 (3.3)	15 (0.9)	37 (2.1)	40 (2.2)	113 (5.8)	264 (2.9)
2nd	1285 (72.8)	1269 (75.3)	1279 (71.7)	1239 (67.2)	1261 (65.0)	6333 (70.2)
3rd	421 (23.9)	401 (23.8)	469 (26.3)	564 (30.6)	567 (29.2)	2422 (26.9)
Pregnancy trimester during follow‐up						
1st	‐	‐	‐	2 (0.1)	1 (0.1)	3 (0.0)
2nd	18 (1.0)	64 (3.9)	8 (0.5)	14 (0.8)	55 (2.9)	159 (1.8)
3rd	1709 (99.0)	1583 (96.1)	1724 (99.5)	1781 (99.1)	1834 (97.0)	8631 (98.2)

^a^ The wealth index is derived from the information on household ownership of durable goods and its housing characteristics.

The median age of pregnant women was 22 years (interquartile range [IQR] = 20–26), and 33% of women were nulliparous women. Most women (96%) were homemakers, and 98% were in their third trimester when the follow‐up began. The mean follow‐up time for a pregnant woman was 12.5 weeks (±SD, 6.4) during the influenza season. Pregnant women were in their third trimester for 79% of their total follow‐up time.

Among 8963 infants, 5116 (57%) reported at least one ARI episode. The highest number of ARI episodes was reported among 941 (48%) infants in 2017, and the lowest number was reported among 589 (29%) in 2015 (Table 2). Among infants with an ARI (*n* = 3923), 110 (3%) had influenza virus infection detected by rRT‐PCR. The overall incidence of influenza virus infection during peak season among infants per 10,000 infant‐months during 2013–2017 was 21.9 (18.2–26.5) and ranged from 4.9 (95% CI: 1.8–13.9) in 2016 to 45.0 (95% CI: 33.9–59.9) in 2017 (Table 2).

**TABLE 2:** Clinical symptoms, ARI, and influenza virus infection among young infants during the total follow‐up period of five cohorts (July–September 2013 and May–September 2014–2017).

Clinical symptoms, ARI and influenza virus infection	<6‐month infants cohort					
	2013 (*n* = 1660)	2014 (*n* = 1825)	2015 (*n* = 1796)	2016 (*n* = 1843)	2017 (*n* = 1839)	Overall (*n* = 8963)
Total ARI episodes	1103	1176	589	984	1264	5116
ARI cases	798 (46%)	888 (47%)	521 (29%)	775 (42%)	941 (48%)	3923 (44%)
More than 1 ARI	240 (14%)	251 (13%)	63 (4%)	178 (10%)	271 (14%)	1003 (11%)
Reported symptoms						
Fever	387 (23%)	939 (52%)	721 (40%)	992 (54%)	1136 (62%)	4175 (47%)
Cough	399 (24%)	917 (50%)	559 (31%)	647 (35%)	661 (36%)	3183 (36%)
Rhinorrhea	573 (34%)	848 (47%)	855 (48%)	929 (50%)	759 (41%)	3964 (44%)
Difficulty breathing	30 (2%)	140 (8%)	49 (3%)	40 (2%)	34 (2%)	293 (3%)
Influenza virus detected among ARI cases						
Influenza virus	9 (1%)	23 (3%)	27 (5%)	4^a^ (1%)	47^a^ (5%)	110 (2%)
Influenza A(H1N1)pdm09	‐	‐	8 (30%)	‐	2 (4%)	10 (9%)
Influenza A/H3N2	3 (33%)	8 (38%)	13 (48%)	2 (40%)	14 (29%)	40 (36%)
Influenza B/Victoria	5 (11%)	‐	1 (4%)	2 (40%)	17 (35%)	25 (23%)
Influenza B/Yamagata	1 (55%)	13 (62%)	5 (19%)	1 (20%)	15 (31%)	35 (32%)
Incidence of influenza virus infection						
Total infant‐months of follow‐up	9565.4	10601.8	10564.2	10129.4	10657.0	51517.8
Influenza incidence per 10,000 infant‐months during peak season (95% CI)	9.4 (4.9–18.1)	22.6 (15.0–34.2)	25.6 (17.6–37.2)	4.9 (1.8–13.9)	45.0 (33.9–59.9)	21.9 (18.2–26.5)

Abbreviations: ARI, acute respiratory illness; CI, confidence intervals.

^a^ Co‐infection.

**FIGURE 3:**
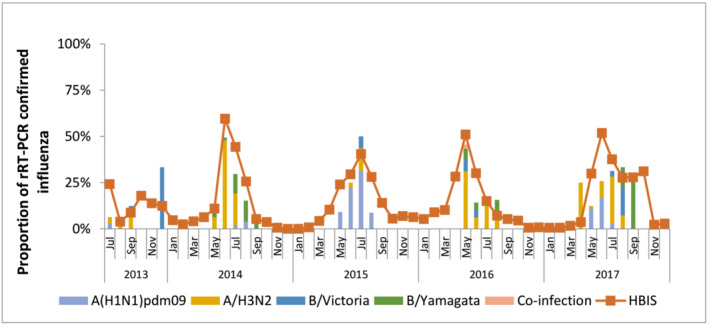
Influenza virus infection among pregnant women in eight sub‐districts of Bangladesh, 2013–2017.

The ILI was reported among pregnant women, and the number of ILI episodes ranged from 247 episodes among 237/1765 (13%) participants in 2013 to 285 episodes and among 273/1685 (16%) participants in 2014. Forty‐five (0.5%) of all pregnant women reported more than one ILI episode (Table 3). We found that 98% of illness episodes among pregnant women were in the third trimester of pregnancy. The mean time from illness onset to swab collection was 3.8 (SD: 4.8) days. Among pregnant women with ILI (*n* = 1193), 276 (23%) had influenza virus infection detected by rRT‐PCR. Among pregnant women enrolled in each cohort, the per cent positivity of influenza was highest in 2014 (6.5%) and was lowest in 2013 (0.5%). The incidence of influenza virus infection was 158.5/10000 pregnant women‐months (95% CI: 141.4–177.6) and ranged from the highest of 1176.6/10000 pregnant women‐months (95% CI: 1009.1‐1371.9) in 2014, and the lowest 17.8/10000 pregnant women‐months (95% CI: 8.9–35.6) in 2013 (Table 3). None of the pregnant women was hospitalized for their ILI or influenza virus infection. The circulating strains are listed in Table 3, and their findings are consistent with the hospital‐based influenza surveillance (HBIS) in Bangladesh (Figure 3). Influenza virus infection was rare outside the influenza season (Figure 3).

**TABLE 3:** Clinical symptoms, ILI, and influenza virus infection among pregnant women during the total follow‐up period of five cohorts (July–December 2013 and May–December 2014–2017).

Clinical symptoms, ILI and influenza virus infection	Pregnant women cohort					
	2013 (*n* = 1765)	2014 (*n* = 1685)	2015 (*n* = 1786)	2016 (*n* = 1843)	2017 (*n* = 1941)	Overall (*n* = 9020)
Total ILI episodes	247	285	183	184	342	1241
ILI participants	237 (13%)	273 (16%)	179 (10%)	179 (10%)	325 (17%)	1193 (13%)
More than one ILI	9 (0.5%)	12 (0.7%)	4 (0.2%)	5 (0.3%)	15 (0.8%)	45 (0.5%)
Reported symptoms						
Fever	237 (13%)	273 (16%)	179 (10%)	179 (10%)	325 (17%)	1193 (13%)
Cough	237 (13%)	273 (16%)	179 (10%)	179 (10%)	325 (17%)	1193 (13%)
Rhinorrhea	192 (11%)	230 (14%)	141 (8%)	147 (8%)	278 (14%)	988 (11%)
Sore throat	73 (4%)	36(2%)	27 (2%)	38 (2%)	97 (5%)	271 (3%)
Difficulty breathing	45 (3%)	23 (1%)	7 (0.4%)	2 (0.1%)	11 (1%)	88 (1%)
Influenza virus detected among ILI cases						
Influenza virus	8 (1.2%)	109 (38%)	31^a^ (17%)	56^a^ (30%)	76 (22%)	280 (23%)
Influenza A(H1N1)pdm09	2 (25%)	1 (1%)	30 (97%)	0	33 (43%)	66 (24%)
Influenza A/H3N2	4(50%)	93 (85%)	4 (13%)	36 (69%)	31 (41%)	168 (60%)
Influenza B/Victoria	2 (25%)	1 (1%)	0	8 (16%)	6 (8%)	17 (6%)
Influenza B/Yamagata	0	12 (11%)	0	9 (25%)	6 (8%)	27 (10%)
Incidence of influenza virus infection						
Total pregnant women‐months of follow‐up	4493.5	4794.0	5543.8	5881.3	5996.7	26709.3
Influenza incidence per 10,000 pregnant women‐months (95% CI)	17.8 (8.9–35.6)	1176.6 (1009.1–1371.9)	55.9 (39.5–79.2)	794.1 (631.6–998.5)	126.7 (101.7–157.8)	158.5 (141.4–177.6)

Abbreviations: CI, confidence intervals; ILI, influenza‐like illness.

^a^ Co‐infection.

We compared the gestational age determined by the LMP with the gestational age determined by the portable ultrasound machine. There were 586 women between 2016 and 2017 who reported gestational age to be <24 weeks and had an ultrasound. Of those, 141 (24%) were >24 weeks of gestation, determined by the ultrasound. We included the remaining 445 women who were <24 weeks gestation, determined by both LMP and ultrasound, to compare the method of gestational age determination. There was a statistically significant (*P* = 0.002) mean difference of 2.2 weeks (95% CI: 2.0–2.4) overestimate of gestational age obtained by date of LMP compared with ultrasound for the same participants.

Among 8963 enrolled infants, the mean birth weight was 2965 (95% CI: 2952‐2977) grams, and LBW was observed in 12.6% of infants (Table 4). There were 161 (1.8%) stillbirths and 112 (1.2%) early neonatal deaths within 7 days of birth.

**TABLE 4:** Pregnancy outcomes of pregnant women in eight sub‐districts of Bangladesh (July–December 2013 and May–December 2014–2017).

Outcomes	Pregnant women cohort					
	2013 (*n* = 1765)	2014 (*n* = 1685)	2015 (*n* = 1786)	2016 (*n* = 1843)	2017 (*n* = 1941)	2013–17 (*n* = 9020)
Mean birth weight grams (95% CI)	3016 (2983–3050)	2993 (2966–3020)	2925 (2900–2951)	2950 (2925–2975)	2939 (2910–2967	2965 (2952–2977)
Missing birth weight data, n (%)	388 (22.0)	134 (7.0)	115 (6.4)	169 (9.2)	701 (35.9)	1507 (16.3)
Low birth weight, n (%)	209 (15.2)	336 (18.0)	239 (14.2)	218 (13.0)	163 (13.0)	1165 (14.8)
Still birth, n (%)	47 (2.7)	53 (2.8)	0	0	61 (3.2)	161 (1.8)
Death within 7 days of birth, n (%)	25 (1.4)	29 (1.5)	8 (0.5)	17 (0.9)	33 (1.7)	112 (1.2)

## 4 DISCUSSION

Our study was a resource‐intense effort to document the incidence of influenza among pregnant women for the first time in Bangladesh. We followed five separate cohorts of unvaccinated pregnant women prospectively through delivery and their infants until 6 months of age during the influenza seasons of 2013 to 2017. There was a notable occurrence of ILI episodes among pregnant women and ARI episodes among infants. The estimated incidence of influenza among pregnant women and infants was the highest in 2014 and 2017 when the predominant circulating viruses were Influenza A/H3N2 and A(H1N1)pdm09, respectively. A community middle‐income based multicountry study reported a weighted overall influenza incidence of 88.7/10000 pregnant women‐months.^20^ From this multicounty study, neighboring countries of Bangladesh, firstly, India had an influenza IR of 90.3/10000 pregnant women‐months and secondly, Thailand had 117.7/10000 pregnant women‐months in 2017.^20^ Our findings during 2017 (126.7/10000 pregnant women‐months) were higher than that of the estimates of Thailand, and our cumulative estimates over 5 years were higher (158.5/10000 pregnant women months) than those reported in neighboring countries.

Overall, our findings on predominant circulating subtypes were consistent with routine hospital‐based surveillance for the same areas in Bangladesh. There was consistency with the circulating subtypes identified through routine surveillance.^21^ However, during the 2013 and 2017 influenza seasons, when the incidence of influenza virus infection among infants was high, the predominant subtypes were Influenza B/Yamagata and B/Victoria, respectively. This finding was inconsistent with local routine surveillance data, which demonstrated Influenza A viruses predominated during the influenza seasons of 2013 and 2017.^21^ Further, while influenza virus infections can occur outside of the typical circulation period in settings such as the United States^22^ and Australia,^23^ we did not observe many influenza virus infections outside of the regular influenza season in Bangladesh.

The majority of pregnant women cohort follow‐up time happened during the third trimester, and 98% of influenza virus infections occurred during the third trimester. There are heightened risks of fetal death and other adverse birth outcomes because of maternal seasonal influenza infection.^24^ The US CDC Advisory Committee on Immunization Practices and the American College of Obstetricians and Gynecologists have recommended influenza vaccination to women in any trimester since 2004.^25^ Information on epidemics of influenza virus infection could be critical to determining maternal vaccine policy, as there is a recent evidence regarding the trade‐off regarding the timing of vaccination. Vaccinating earlier in pregnancy results in a more significant proportion of pregnancy, while vaccinating later in pregnancy confers more protection to the newborn by transplacental antibodies transfer.^26^

Our study did not document any hospitalization among pregnant women for influenza illnesses. A multi‐country study reported a weighted incidence of influenza associated hospitalization of 1.6–2.2 per 10,000 pregnant women months during 2017–2018 seasons.^20^ In our study, limited influenza virus infection during early pregnancy, together with no hospitalizations, may be attributable to less severity because of infection and minimal rare adverse outcomes during pregnancy.^24^ A multicountry study over multiple influenza seasons^27^ have reported that severe complications were more likely to be found among hospitalized cases with influenza virus infection in several different studies compared to non‐hospitalized cases.^28^ Findings of no hospitalization and no adverse outcomes are suggestive that influenza was not severe. However, these were not our study objectives, and the sample size was not powered enough to determine hospitalizations and adverse outcomes.

As there was concern about the accuracy of measuring gestational age using the date of the LMP because of potential recall bias and the difficulty of predicting gestational age by menstrual history,^29^ we explored the feasibility of using portable ultrasound machines. We observed a statistically significant difference in gestational age determined by ultrasound compared with the date of the LMP, with the latter typically overestimating gestational age. This finding suggests that the use of LMP may underestimate the prevalence of preterm birth and, in turn, may result in measurement errors in studies evaluating the effect of influenza virus infections on preterm birth. Ultrasound methods are more reliable for determining gestational age.^16^, ^29^ However, the availability and feasibility of ultrasound machines and trained and experienced sonologist in low‐resource settings are limited. However, our findings suggests that with resources available, it is possible to perform this diagnostic measure in a low‐resource community setting to record more accurate data.

The strengths of our study included recruiting five large cohorts comprising 9020 pregnant women and following them for over five consecutive influenza seasons. They were actively followed up over the influenza season, and we also followed up on the infants born to them until 6 months of age. Despite these strengths, our study has some limitations that demand discussion. We enrolled pregnant women and infants from four districts of Bangladesh, representing approximately 10% of the population^30^; thus, our estimates may not be generalizable to the entire country or other countries. However, these districts are geographically representative and likely represent pregnant women's age distribution in Bangladesh. Another limitation was the delayed start of the enrollment and follow‐up in 2013, which may have led to an underestimation of influenza virus infections among pregnant women and infants. We could not estimate the incidence of influenza virus infections earlier in pregnancy as most of the infections were in the third trimester. Another limitation may be minimal influenza exposure among infants <6 months. Most infants (58%) were born towards the end of the typical influenza circulation period. As influenza virus infections outside of the typical season are not common in Bangladesh,^21^ this may have underestimated influenza virus infection among infants. Severe influenza infection and subsequent adverse pregnancy and fetal outcomes remain crucial evidences required for national policy driven decisions of influenza control measures among this high priority population. Our study was not powered enough to capture severe influenza cases that may lead to adverse pregnancy and fetal outcomes.

Despite these limitations, our study provides the most comprehensive influenza illness estimates and evidence of the burden of influenza on pregnant women and infants <6 months of age in Bangladesh. Influenza virus infection among pregnant women and infants was common in Bangladesh. These findings may be valuable to inform policy decisions about possible prevention and control measures for these high priority groups in Bangladesh, such as the use of influenza vaccine in this risk group and the availability of antivirals to treat influenza virus infections among infants.

## AUTHOR CONTRIBUTIONS

**Zubair Akhtar:** Data curation; formal analysis; funding acquisition; methodology; project administration; supervision; writing—original draft; writing—review and editing. **Probir Ghosh:** Data curation; formal analysis. **Mejbah Bhuiyan:** Conceptualization; investigation; project administration. **Katharine Sturm‐Ramirez:** Conceptualization; funding acquisition; writing—review and editing. **Mohammed Rahman:** Investigation; writing—review and editing. **Md. Howlader:** Project administration. **Fatimah Dawood:** Writing—review and editing. **Fahmida Chowdhury:** Funding acquisition; investigation; methodology; project administration; supervision; writing—review and editing. **Danielle Iuliano:** Conceptualization; funding acquisition; supervision; writing—review and editing.

## CONFLICT OF INTEREST STATEMENT

The authors have no competing interest to declare.

### PEER REVIEW

The peer review history for this article is available at https://www.webofscience.com/api/gateway/wos/peer-review/10.1111/irv.13175.

## ETHICS APPROVAL

The study was approved by the icddr,b institutional review board (IRB) before enrolling participants. Centers for Disease Control and Prevention (CDC)'s Human Research Protection Office has reviewed and approved reliance on icddr,b's IRB. Written informed consent to participate in the study was obtained from the participant or the infant's mother before enrolling, data collection, or specimen collection.

## DISCLAIMER

The findings and conclusions in this report are those of the author(s) and do not necessarily represent the official position of the Centers for Disease Control and Prevention (CDC).

## Data Availability

Data generated during the study are subject to a data access policy of icddr,b and are available from icddr,b's research administration on reasonable request through the corresponding author.
